# Cross-talk between macrophages and gut microbiota in inflammatory bowel disease: a dynamic interplay influencing pathogenesis and therapy

**DOI:** 10.3389/fmed.2024.1457218

**Published:** 2024-09-16

**Authors:** Shiyang Ning, Zhe Zhang, Chuan Zhou, Binbin Wang, Zhanju Liu, Baisui Feng

**Affiliations:** ^1^Department of Gastroenterology, The Second Affiliated Hospital of Zhengzhou University, Zhengzhou, China; ^2^Department of Gastroenterology, The Shanghai Tenth People’s Hospital, Shanghai, China

**Keywords:** IBD, gut microbiota, macrophage, SCFAs, TRP, secondary bile acid

## Abstract

Inflammatory bowel disease (IBD), which includes ulcerative colitis (UC) and Crohn’s disease (CD), is a group of chronic immune-mediated gastrointestinal disorders. The etiology of IBD is multifactorial, involving genetic susceptibility, environmental factors, and a complex interplay between the gut microbiota and the host’s immune system. Intestinal resident macrophages play an important role in the pathogenesis and progress of IBD, as well as in maintaining intestinal homeostasis and facilitating tissue repair. This review delves into the intricate relationship between intestinal macrophages and gut microbiota, highlighting their pivotal roles in IBD pathogenesis. We discuss the impact of macrophage dysregulation and the consequent polarization of different phenotypes on intestinal inflammation. Furthermore, we explore the compositional and functional alterations in gut microbiota associated with IBD, including the emerging significance of fungal and viral components. This review also examines the effects of current therapeutic strategies, such as 5-aminosalicylic acid (5-ASA), antibiotics, steroids, immunomodulators, and biologics, on gut microbiota and macrophage function. We underscore the potential of fecal microbiota transplantation (FMT) and probiotics as innovative approaches to modulate the gut microbiome in IBD. The aim is to provide insights into the development of novel therapies targeting the gut microbiota and macrophages to improve IBD management.

## 1 Introduction

Inflammatory bowel disease (IBD) is a group of non-specific chronic gastrointestinal inflammatory diseases mainly mediated by immunity. The incidence of IBD is increasing worldwide, including in both developed and developing countries, as well as in China (1, 2). IBD includes two types: ulcerative colitis (UC) and Crohn’s disease (CD), the pathogenesis of which is not yet clear. Currently, no treatment can completely cure IBD; existing treatments can only temporarily achieve remission for a short period rather than providing a complete cure. It is well known that the development of IBD is related to genetic susceptibility and environmental factors. People living in different countries or regions have significant differences in the composition of their intestinal microbiota (3). The changing pattern of intestinal microbiota in Chinese and Western IBD patients is similar, which suggests that the disturbance of intestinal microbiota plays an important role in the pathogenesis of IBD (4, 5).

Macrophages play a crucial role as innate immune cells in the human body, maintaining homeostasis by engulfing aging cells and debris, immune complexes, bacteria, and other waste products. The disturbance of intestinal macrophages is a significant feature of inflammatory bowel disease (IBD). Macrophages are highly sensitive to changes in the intestinal tissue and microenvironment. In the progression of IBD, the interaction between intestinal resident macrophages and gut microbiota plays a key role. This interaction leads to changes in macrophage function and induces polarization to different phenotypes, which becomes pivotal in the development of inflammation (6). Due to the significant role of macrophages’ interaction with gut microbiota in IBD, they are considered promising therapeutic targets for this condition. This review explores how these two factors influence each other in the occurrence and development of IBD, as well as how existing therapeutic methods impact both. It is hoped that this exploration will lead to the development of new therapies for IBD targeting intestinal flora and macrophages.

## 2 Gut microbiota in IBD

The relationship between the disturbance of gut microbiota and IBD is not a simple causal one, but rather a complex and dynamic one (7). It is widely accepted that the imbalance of gut microbiota composition and diversity plays an important role in the development of IBD (8). In healthy individuals, the dominant bacterial phyla include *Firmicutes*, *Bacteroidetes*, *Proteobacteria*, and *Actinobacteria*, which collectively account for more than 90% of the total. Specifically, *Firmicutes* and *Bacteroidetes* are predominant (9–11).

Individuals with inflammatory bowel disease (IBD) exhibit significant alterations in the composition of their gut microbiota. In comparison to the healthy control group, findings indicate a relative increase in *Actinobacteria* and *Proteobacteria* (*Enterobacteriaceae*), while *Firmicutes* (*Clostridium*) and *Bacteroidetes* show a relative decrease. Additionally, there is an observed rise in potentially pathogenic bacteria such as *Escherichia coli*, *Klebsiella pneumoniae*, and *Neisseria*. A systematic review has revealed that IBD patients’ intestinal flora is characterized by a reduction in the abundance of *Coriobacteriaceae* and *Faecalibacterium prausnitzii*. Furthermore, patients with Crohn’s disease demonstrate increased levels of *Actinomyces*, *Christensenellaceae*, *Veillonella*, and *Escherichia coli*. Conversely, ulcerative colitis patients display decreased levels of *Eubacterium rectale* and *Akkermansia* but increased levels of *E coli* (12). This significant alteration in the microbiota of patients with inflammatory bowel disease (IBD) is referred to as gut microbiota disorder. These changes are closely associated with the onset and severity of IBD. For instance, *E. coli* and *K. pneumoniae* isolated from patients with Crohn’s disease (CD), as well as *F. varium* isolated from patients with ulcerative colitis (UC), have been demonstrated to independently induce the development and progression of experimental colitis. Following the transplantation of fecal bacteria from patients with Crohn’s ileocolitis (CD_L3) into germ-free mice orally, the mouse model consistently exhibited characteristics resembling those observed in Crohn’s disease patients. These features included a non-continuous pattern of colitis, localization primarily in the proximal colon, and enlarged isolated lymphoid follicles and/or tertiary lymphoid organ neogenesis, and a CD-like transcriptomic pattern. Notably, these manifestations were not observed in the ileum of these mice (13). The current hypothesis suggests that a decrease in *Firmicutes* serves as a protective factor for IBD, whereas an increase in *Proteobacteria* acts as an invasive factor (5, 14). In addition to alterations in microbial composition, the intestinal microbiota diversity in individuals with inflammatory bowel disease (IBD) exhibited a significant reduction compared to healthy controls, as evidenced by markedly lower species richness, Shannon index, and Simpson index than those observed in healthy individuals. Healthy individuals had approximately twice the microbial diversity compared to IBD patients (15).

In the human gut, in addition to the bacterial group, there are also a variety of fungal colonies and viruses, known as the fungus group and the virus group. Until now, most studies related to the intestinal microbiota of IBD have primarily focused on bacteria. However, other members of the intestinal microbiome such as fungi, bacteriophages, and archaea also play a role in the pathogenesis of human IBD. Sokol H and colleagues utilized ITS2 sequencing to analyze the fungal composition in the fecal microbiota of 235 patients with inflammatory bowel disease (IBD) and compared it with that of 38 healthy subjects (HS). The study revealed that, unlike the HS group, the fecal content of *Saccharomyces cerevisiae* was reduced, while the presence of *Candida albicans* was elevated in IBD patients. These findings suggest that, in addition to bacteria, fungi may also play a significant role in the pathogenesis of IBD (16). Dysregulation of the enterovirus group, driven primarily by the amplification of Caudovirales bacteriophages, is associated with the development of inflammatory bowel disease (IBD) (17). Additionally, numerous individual eukaryotic viruses have been reported to be associated with IBD; however, the underlying mechanisms remain unknown and require further investigation (18).

## 3 Macrophage in IBD

**FIGURE 1:**
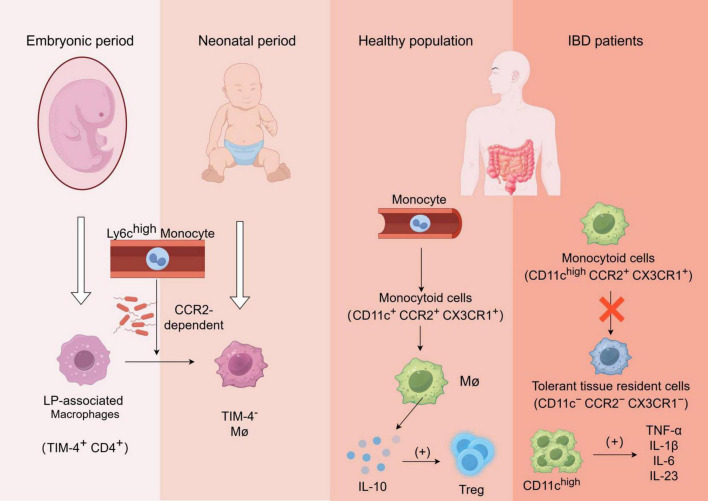
Formation and function of gut-resident macrophages and alterations in macrophage functionality among patients with inflammatory bowel disease (IBD): The laminae propria-associated macrophages (TIM-4^+^ CD4^+^) present in the embryo are rapidly replaced by Ly6c^high^ monocyte-derived TIM-4^–^ macrophages after birth, in a manner dependent on CCR2 and gut microbiota;Monocytes in the bloodstream of healthy individuals undergo differentiation into monocytoid cells(CD11c^+^ CCR2^+^ CX3CR1^+^), which subsequently mature into macrophages and secrete IL-10 to regulate Treg cells;In patients with inflammatory bowel disease (IBD), the transformation process of monocyte-like cells into tolerant tissue-resident cells is impaired, leading to the production of various pro-inflammatory cytokines by aggregated CD11c^high^ cells, which further recruit other immune cells and exacerbate inflammation.

In recent decades, the prevailing belief regarding macrophages was that they were a type of mononuclear cells derived from bone marrow hematopoietic stem cells, primarily responsible for phagocytic functions in the human body (Figure 1). However, advancements in science and technology have revealed through techniques such as single-cell RNA sequencing (scRNA-seq) and fate-mapping that macrophages originate early in embryogenesis, colonize various organs, and differentiate into tissue-resident macrophages (TRMs). These TRMs exhibit high self-renewal capabilities independent of input from bone-marrow hematopoietic stem cells (HSCs) (19).

Macrophages are distributed throughout the entire gastrointestinal tract at various levels. Based on their anatomical location, intestinal macrophages can be classified into lamina propria macrophages (LpMs), submucosal macrophages, and muscularis macrophages (MMs). Among these subsets, LpMs exhibit a higher abundance and distinct profiles of cytokine and chemokine expression, which underlie their diverse functions. The intestinal mucosa efficiently eliminates foreign pathogens and toxins while tolerating food molecules and gut microbes. LpMs play a crucial role in maintaining intestinal homeostasis by continuously phagocytosing apoptotic cells (20). LpMs primarily consist of two subsets: pro-inflammatory ones located closer to the epithelial surface with high antigen presentation capacity and phagocytic activity (21).

During fetal development, lamina-associated macrophages (TIM-4^+^CD4^+^ long-lived macrophages) are present but quickly replaced by short-lived monocyte-derived TIM-4^–^macrophages shortly after birth via recruitment of circulating Ly6C^high^ monocytes in a CCR2-dependent manner. In adulthood, these populations of macrophages undergo replenishment which relies on microbiota involvement. Moreover, newly recruited Ly6C^high^ monocytes have the ability to differentiate into tolerant macrophages (Ly6C^–^MHCII^high^CX3CR1^high^CCR2^–^), influenced by both “monocyte waterfall” intermediates and the local microenvironment (22, 23).

Extensive clusters of neuronal cells are observed in the submucosa and muscle layers, while muscular macrophages (MMs) exhibit two distinct differentiation trajectories: one that upregulates genes associated with immune activation and angiogenesis, and another that upregulates genes related to neuronal homeostasis. Depletion of MMs may lead to the loss of intestinal neurons, increased vascular permeability, diminished intestinal motility, and impaired secretion (22, 24).

Intestinal macrophages are subjected to more extensive exposure to the intestinal microbiota and/or their derivatives in comparison to other tissue-resident macrophages, necessitating a continuous replenishment process. The rate of replenishment is influenced by the diversity of the intestinal microbiota, potentially leading to an accelerated recruitment of macrophages (25).

In healthy individuals, blood-derived monocytes have the capacity to differentiate into CD11c^+^CCR2^+^CX3CR1^+^ monocyte-like cells, which subsequently undergo maturation into macrophages and secrete IL-10 to facilitate regulatory T-cell expansion. However, in active inflammatory bowel disease (IBD), the conversion process of pro-inflammatory monocyte-like cells cells (CD11c^high^CCR2^+^CX3CR1^+^) into tolerant tissues (CD11c^–^CCR2^–^CX3CR1^–^) is impeded. Consequently, there is an accumulation of CD11c^high^ monocyte-like cells along with a substantial production of pro-inflammatory cytokines upon exposure to symbiotic bacteria, including TNF-α, IL-1β, IL-6, and IL-23. These cytokines further activate and recruit other immune cells to the site of inflammation, thereby exacerbating intestinal inflammation (26, 27).

Macrophages display a diverse array of phenotypes within the human body. Monocytes are stimulated by M-CSF (macrophage colony-stimulating factor) to differentiate into M0 macrophages. Upon exposure to various stimuli, M0 macrophages undergo distinct polarization states. These polarized macrophages can be categorized into two primary types: classically activated M1-like macrophages and alternatively activated M2-like macrophages (28). Moreover, M2-like macrophages can be further classified into four distinct subgroups: M2a, associated with tissue repair and anti-inflammatory responses; proinflammatory or anti-inflammatory-associated M2b; tissue remodeling-associated M2c; and tumor progression and angiogenesis-promoting M2d (29). M1-like macrophages are typically induced by pro-inflammatory Th1 cytokines such as IFN-γ and TNF-α, as well as microbial factors. Conversely, Th2 cytokines (such as IL-4 and IL-13), anti-inflammatory cytokines (such as IL-10 and TGF-β), or other stimuli can induce the differentiation of M2 macrophages. The current studies indicate a strong association between macrophage polarization and signal transducer and activator of transcription family (STATs), peroxisome proliferator-activated receptor (PPAR), cAMP response element binding protein (CREB)-CCAAT/enhancer binding protein (C/EBP), hypoxia-inducible factors (HIF), nuclear factor kappa B (NFκB) and interferon regulatory factors (IRF). Among these factors, STAT-1 and NF-κB play crucial roles in the M1-like phenotype polarization of macrophages induced by IFN-γ or LPS stimulation, while IRF5 also significantly contributes to this process. Transcription factors including STAT-6, PPAR-γ, and C/EBP-β are important for M2 macrophage polarization. In patients with inflammatory bowel disease (IBD), there is an increased ratio of M1/M2 macrophages. M1-like macrophages directly contribute to epithelial barrier breakdown through mediating epithelial apoptosis and dysregulation of tight junctions, whereas M2-like macrophages are considered potential contributors to mucosal tolerance reconstruction and repair during IBD immunotherapy (29).

## 4 The interaction between macrophages and microbiota is crucial in the development of inflammatory bowel disease

**FIGURE 2:**
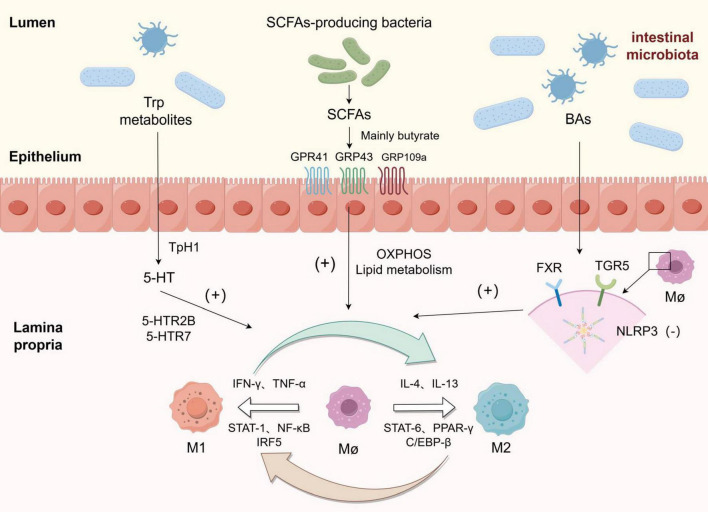
Macrophages were polarized towards M1 and M2 phenotypes in response to various stimuli, and the metabolites derived from gut microbiota could enhance the polarization of macrophages towards the M2 phenotype: Among tryptophan metabolites, 5-HT upregulated the expression of genes associated with M2 polarization by binding to 5-HTR2B/5-HTR7 receptors. Short-chain fatty acids (SCFAs) exert their effects on the intestine through GRP41, GRP43, and GRP109a receptors, while butyrate and other SCFAs promote polarization by reprogramming macrophage metabolism towards oxidative phosphorylation (OXPHOS) and lipid metabolism pathways. Bile acids (BAs) inhibit NLRP3 inflammasome activation by binding to FXR and TGR receptors on macrophages, thereby inducing polarization towards an M2 phenotype.

The intestinal microbiota primarily modulates the disease process in intestinal macrophages through the production of various metabolites, such as short-chain fatty acids, secondary bile acids, and indole products (Figure 2).

### 4.1 Short-chain fatty acids (SCFAs)

Short-chain fatty acids are a group of fatty acids characterized by their abbreviated carbon chains, which are synthesized in the human gut by intestinal flora through the fermentation of indigestible carbohydrates (such as dietary fiber and resistant starch). This category primarily encompasses formic acid, acetic acid, propionic acid, butyric acid, and valeric acid. Acetic acid can be generated from pyruvate via the acetyl-CoA and Wood-Ljungdahl pathways. Propionic acid is predominantly derived from succinic acid through the succinic acid pathway or from lactic acid via the acrylate pathway. Butyric acid is formed through various processes involving acetyl-CoA and butyryl CoA, as well as acetic and lactic acids (30, 31). The predominant proportion of intestinal short-chain fatty acids (SCFAs) was accounted for by acetic acid, followed by propionic acid and butyric acid, with a ratio of approximately 3:1:1. These three SCFAs collectively constituted over 95% of the total SCFAs content (32, 33). Several factors influence SCFA production including gut transit time of food, diversity, and absolute abundance of gut microbiota, with specific microbial species playing crucial roles in their synthesis (34). In previous studies, the group of intestinal microbiota capable of specifically producing short-chain fatty acids has been collectively referred to as SCFAs-producing bacteria. Previously identified strains such as *Akkermansia municiphilla*, *Faecalibacterium prausnitzii*, *Eubacterium rectale*, *Eubacterium hallii*, and *Ruminococcus bromii* have demonstrated their ability to produce SCFAs (35). Notably, *Ruminococcus bromii* exhibits a significant enhancement in butyric acid production through resistant starch fermentation in the colon (36). Furthermore, research has shown a substantial reduction in the abundance of SCFAs-producing bacterium *Faecalibacterium prausnitzii* and other bacterial species within the colon flora of patients with inflammatory bowel disease (37).

SCFAs may impact intestinal and host metabolism by activating G protein-coupled cell surface receptors such as GPR41 (also known as free fatty acid receptor 3), GPR43 (also known as free fatty acid receptor 2), and GPR109a. These SCFAs produced by gut flora are distributed throughout the body via blood circulation and play a crucial role in autoimmunity: unlike medium-chain fatty acids (MCFAs) and long-chain fatty acids (LCFAs), SCFAs possess anti-inflammatory properties by promoting differentiation of initial CD4^+^ T cells into regulatory T cells rather than Th1 or Th17 cells (38). Among the various short-chain fatty acids (SCFAs), extensive research has been conducted on butyric acid, which has demonstrated its potential to modulate beneficial effects in the colon. Specifically, it exerts inhibitory effects on histone deacetylases (HDACs) that play a crucial role in promoting cell differentiation, suppressing colon inflammation, and inducing cell cycle arrest and apoptosis of colon cancer cells (39).

In addition to modulating CD4^+^ T cell differentiation and inhibiting inflammation, short-chain fatty acids (SCFAs) also exert regulatory effects on macrophage function. Specifically, butyrate has been demonstrated to significantly suppress the production of nitric oxide (NO), a key antimicrobial effector, as well as proinflammatory cytokines IL-6 and IL-12p40 in bone marrow-derived macrophages. Treatment with butyrate leads to reduced transcription levels of Il6, Nos2, Il12a, and Il12b genes while having no impact on Tnfa and Ccl2 expression - the major response genes encoding TNF-α and MCP-1 respectively. These anti-inflammatory effects are mediated by butyrate through inhibition of the histone deacetylase pathway independent of G protein-coupled receptors (40). M2-like macrophages exhibit a complete tricarboxylic acid (TCA) cycle that relies on oxidative phosphorylation (OXPHOS) (41). However, the presence of butyric acid produced by SCFA-producing bacteria can reprogram macrophage metabolism towards oxidative phosphorylation (OXPHOS) and lipid metabolism, thereby promoting polarization towards the anti-inflammatory M2-like phenotype. Recently, Cao et al. developed a prodrug called SerBut by combining butyric acid with serine. Oral administration of SerBut significantly elevates butyric acid levels in mice while also increasing regulatory T cells and reducing the proportion of M1-like macrophages to modulate immune responses. As a promising therapeutic agent for autoimmune and inflammatory diseases, SerBut may potentially play a role in future IBD treatment (42).

### 4.2 Secondary bile acids

The primary bile acids, cholic acid (CA) and glycochenodeoxycholic acid (GCDCA) are synthesized by hepatocytes from cholesterol. These bile acids can be conjugated with glycine (approximately 90%) or taurine, collectively referred to as primary bile acids (43). Following *de novo* synthesis and binding, the primary bile acids enter the hepatic duct through the hepatic bile duct and subsequently transit through the common bile duct into the gallbladder. Eventually, they reach the duodenum via the Oddi sphincter where they play a pivotal role in facilitating digestion and absorption of fat-soluble nutrients while also serving as substrates for various bacterial enzyme metabolism (44). Conjugated bile acids are deconjugated via the removal of glycine or taurine groups, subsequently undergoing conversion into secondary bile acids such as deoxycholic acid (DCA) and lithocholic acid (LCA) (45). The transformation from primary to secondary bile acids is primarily mediated by the predominant bacterial genus *Bacteroides*. In patients with inflammatory bowel disease (IBD), there is a significant reduction in *Bifidobacteria* and *Lactobacillus* species (46), resulting in a notable decrease in the overall bile acid pool and an elevated ratio of glycine-taurine conjugates specifically observed in Crohn’s disease patients.

Bile acid-activated receptors (BARS) are a class of receptors that specifically bind and interact with bile acids as ligands in humans, including the farnesoid X receptor (FXR, NR1H4), G protein-coupled bile acid receptor 1 (GPBAR-1/TGR5), pregnane X receptor (PXR), vitamin D receptor (VDR), sphingosine 1-phosphate receptor 2 (S1PR2), and retinoic acid-related orphan nuclear hormone receptor gamma t (RORγt). The expression and functionality of these bile acid-activated receptors are highly dependent on the regulation of intestinal microbiota and are negatively modulated by intestinal inflammation. Bile acids activate these receptors to exert their immunomodulatory effects (47). Among them, FXR and TGR5 represent the most prominent members.

FXR, a ligand-activated transcription factor belonging to the nuclear receptor superfamily, has recently emerged as a crucial regulator of cellular inflammation and immune response. In addition to its predominant expression in the ileal epithelium responsible for bile acid absorption, FXR is also expressed in intestinal lamina proper (LP) resident macrophages (48). Upon activation, FXR effectively suppresses the production of pro-inflammatory cytokines such as IL-1β in mouse colon cells and *in vitro* immune cell lines. Furthermore, it attenuates TNF-α production in human immune cells (49). The activation of FXR exerts inhibitory effects on enteric macrophages by antagonizing the NF-κB pathway and reducing the synthesis of pro-inflammatory cytokines and enzymes including IL-1α, IL-1β, IL-6, TNF-α, IFN-γ, cyclooxygenase 1 (COX-1), and cyclooxygenase 2 (COX-2). Notably, its functional resemblance to TGR5 has been reported (50, 51).

TGR5, a member of the G protein-coupled receptor family (GPCRs), exhibits high expression in mononuclear cells/macrophages. Activation of TGR5 in macrophages can counteract CD14/TLR4 activity, thereby attenuating phagocytosis and suppressing the secretion of pro-inflammatory cytokines. BAR501, a selective agonist of TGR5, has the ability to reverse the effects induced by LPS/IFN-γ stimulation on macrophages, leading to a shift in their polarization from the M1-like phenotype to the M2-like phenotype *in vitro* and ameliorating inflammation (52). Nod-like receptor 3 (NLRP3) inflammasome is a complex composed of multiple proteins expressed in neutrophils, macrophages, and lymphocytes. Its assembly facilitates caspase-1 activation and triggers maturation as well as secretion of inflammatory mediators such as interleukin (IL)-1β and IL-18, thus mediating the occurrence and progression of inflammatory response. Previous studies have demonstrated that NLRP3 is associated with susceptibility to inflammatory bowel disease (IBD) while also regulating intestinal homeostasis through control over intestinal epithelial integrity, microbiota composition, and immune response (53, 54). By inhibiting the activation of NLRP3 inflammasome, TGR5 promotes macrophage polarization towards the M2-like phenotype for reducing inflammation (55).

### 4.3 Tryptophan (TRP) metabolites

Tryptophan (TRP) is an essential aromatic amino acid, and its association with intestinal flora and inflammatory bowel disease (IBD) has been demonstrated in numerous studies. Tryptophan is not endogenously synthesized but primarily obtained through dietary intake. There are three principal metabolic pathways of TRP within the human body (56).

(1) Gut microbiota directly convert tryptophan (TRP) into ligand molecules of various aromatic hydrocarbon receptors (AhR), including indole, indole-3-acrylic acid (IA), indole-3-acetic acid (IAA), indole-3-formaldehyde (ICAld), indole-3-propionic acid (IPA), and indole-3-propionic acid (IAA). Additionally, they produce indole-3-lactic acid (ILA), indole-3-acetonitrile (IACN), indole-3-ethanol (IE), and indole-3-carboxylic acid (ICA). Caspase recruitment domain family member 9(CARD9) is one of the susceptibility genes for inflammatory bowel disease. TRP metabolites generated through intestinal flora metabolism play a protective role in the inflammatory response by regulating IL-22 production via AhR binding, which relies on CARD9 expression. In Card^–/–^ mice, gut microbiota fail to metabolize TRP into AhR ligands (57). A recent study demonstrated that Indole-3-acetic acid(IAA) may alleviate DSS-induced colitis in mice by increasing the abundance of Bifidobacterium pseudolongum and promoting its production of R-equol to enhance Foxp3^+^T cell levels (58).

(2) The kynurenine pathway (KP), mediated by indoleamine 2,3-dioxygenase 1 (IDO1) in epithelial cells and immune cells, plays a pivotal role in various signaling pathways. IDO1-mediated tryptophan catabolism leads to alterations in serum KYN/TRP levels, subsequently impacting AhR signaling, GCN2, and mTOR signaling pathways. Within the IDO1/AhR signaling pathway, IDO1 activates tryptophan catabolism to generate the primary metabolite KYN. This metabolite acts as a ligand that triggers AhR activation and promotes the expression of cytokines IL-6, IL-22, and IL-17. Moreover, the IDO1/GCN2 signaling pathway modulates macrophages to produce anti-inflammatory cytokines such as IL-10 and TGF-β for inflammation regulation (59, 60). Furthermore, xanthurenic acid produced by the KP pathway along with XANA and kynurenic acid (KYNA) can activate AhR and IL-22 to stimulate intestinal epithelial cell (IEC) proliferation while promoting intestinal mucosal healing (61).

(3) The conversion of tryptophan (TRP) into serotonin (5-hydroxytryptamine 5-HT) via the 5-HT pathway, mediated by TRP hydroxylase 1 (TpH1) in enterochromaffin cells, accounts for approximately 1–2% of TRP. This enzymatic process triggers the synthesis of pro-inflammatory cytokines and chemokines through specific receptor binding on immune cells, thereby regulating the inflammatory response (62). In macrophages, serotonin can induce the production of two cytokines, IL-1 and IL-6, through activation of 5HTR-1. Furthermore, 5-HT up-regulates the expression of genes associated with M2-like polarization (such as SERPINB2, THBS1, STAB1, and COL23A1) by binding to 5-HTR2B/5-HTR7 receptors. Additionally, it down-regulates the expression of genes linked to M1 polarization (such as INHBA, CCR2, MMP12, SERPINE1, CD1B, and ALDH1A2), thereby promoting macrophage polarization towards an M2-like phenotype and inhibiting the release of pro-inflammatory cytokines (63, 64).

## 5 Impact of current treatment methods on the gut microbiota and macrophages

Currently, therapeutic drugs for inflammatory bowel disease (IBD) are classified into the following categories: 5-aminosalicylate (5-ASAs), glucocorticoids, immunomodulators, antibiotics, and biologics (such as anti-tumor necrosis factor agents) (65). In addition to pharmacotherapy, a range of emerging microbial-based therapies like fecal microbiota transplantation (FMT), and bacterostatic agents also play a crucial role in clinical management (66). The existing treatments aim to induce and maintain remission in patients while minimizing the adverse effects of the disease without altering or reversing the underlying pathogenic mechanism (67).

### 5.1 Traditional medicine

**(1) 5-ASA**

The classic anti-inflammatory drug mesalazine, also known as 5-aminosalicylic acid (5-ASA), is extensively utilized in the clinical management of inflammatory bowel disease (IBD). Additionally, studies have demonstrated that apart from its therapeutic effects on inflammation, mesalazine also exerts an influence on the intestinal flora by modulating the α diversity and relative abundance of specific bacterial groups such as *Firmicutes* and *Bacteroidetes* in mice without intestinal inflammation. This effect can be vertically transmitted to offspring mice, rendering them less susceptible to colitis induced by dextran sulfate sodium (DSS) and providing a protective effect (68). Furthermore, Schierova et al. illustrated that treatment with 5-ASA for UC patients may enhance the α diversity of their microbiota within a mere two-week period (69). 5-ASA functions as a PPAR agonist, potentially disrupting the nuclear factor (NF)-κB signaling pathway by upregulating PPAR expression and facilitating intranuclear transfer. Consequently, this mechanism may reduce M1-like macrophage infiltration and exerts anti-inflammatory effects (70).

**(2) Antibiotics**

Antibiotic treatment presents a dual challenge in the management of IBD. Patients with IBD may encounter bacterial infections during or after disease activity, and antibiotics can be employed to treat or prevent these infections. However, the use of antibiotics may impact the overall community structure of microbiota in IBD patients, thereby increasing the risk of dysregulation and exacerbation of dysbiosis associated with Crohn’s disease (CD) (71). Dethlefsen et al. (72) demonstrated that broad-spectrum antibiotic usage significantly reduced fecal microbiota, which mostly recovered within 4 weeks after discontinuation; however, some microbial communities remained diminished even after 6 months (72). Moreover, childhood exposure to antibiotics might contribute to the later development of IBD (73). The efficacy of antibiotic treatment for IBD remains uncertain, antibiotic usage can deplete short-chain fatty acids (SCFAs), favoring polarization towards the pro-inflammatory M1-like high-response phenotype in macrophages and leading to excessive production of pro-inflammatory cytokines, ultimately exacerbating intestinal inflammation (74). Consequently, compared to direct administration of antibiotics for treating IBD, their application is more common among CD patients with perianal lesions such as perianal fistula and postoperative Crohn’s disease and bagulitis where metronidazole and ciprofloxacin are clinically utilized (75).

**(3) Corticosteroids (CS)**

Patients with inflammatory bowel disease (IBD) who have developed resistance to 5-aminosalicylic acid (5-ASA) drugs or are unable to tolerate them may opt for steroid hormones as the next step in inducing remission. Treating IBD patients with corticosteroids in a prospective study resulted in changes in the gut microbiota composition; there was an increased abundance of bacteria from the *Blautia*, *Sellimonas*, and unclassified *Ruminococcaceae* families during progression from active to complete remission while *Granulicatella*, *Haemophilus*, and *Streptococcus* decreased (76). The binding of CS to its receptors and its interaction with pro-inflammatory transcription factors, such as NF-κB, may potentially modulate macrophages to exert anti-inflammatory effects (77).

**(4) Immune modulators**

Immunomodulators commonly used for the treatment of inflammatory bowel disease (IBD) include methotrexate, thiopurine, and calcineurin inhibitors. In a study conducted by Yongjun Wang et al., it was concluded that methotrexate did not demonstrate a significant impact on sustaining remission in ulcerative colitis (UC), highlighting the need for more rigorous investigations to evaluate the role of methotrexate in IBD treatment (78). The efficacy of thiopurine in inducing remission is not clearly evident, although it may play a role in maintaining hormone-induced remission in UC (79). Methotrexate utilization has been shown to enhance the clinical remission rate among Crohn’s disease patients who are unresponsive to glucocorticoid therapy (80). Furthermore, the metabolic capacity of the intestinal microbial community, particularly with regard to butyrate synthesis, exhibits close associations with azathioprine (AZA) treatment effectiveness in IBD patients. Therefore, further research is required to investigate the impact of AZA on intestinal flora composition (81). Tacrolimus (TAC) functions as a calcineurin inhibitor, effectively suppressing the activation of NF-κB and MAPK in macrophages. Additionally, it induces apoptosis of macrophages by activating caspases 3 and 9, thereby inhibiting the production of pro-inflammatory cytokines such as IL-12/IL-23 and TNF-α (82).

### 5.2 Innovative therapeutic approach

**(1) Biological agents**

Biologics currently serve as the primary pharmaceutical agents employed in the clinical management of inflammatory bowel disease (IBD), with anti-tumor necrosis factor α (TNF-α) biologics, such as infliximab and adalimumab, being the most commonly utilized. In Murch et al. (83) demonstrated via immunohistochemistry that TNF, a pro-inflammatory cytokine, was overexpressed in the intestinal tissues of patients with Crohn’s disease, leading to the development of anti-TNF-α biologics targeting TNF-α as a potential therapeutic approach for IBD (83). TNF plays a pivotal role in the pathogenesis of IBD by recruiting circulating inflammatory cells to inflamed local tissue sites, inducing inflammation-associated edema and coagulation activation, and contributing to granuloma formation (84). Schierova et al. (85) analyzed bacterial and fungal communities’ diversity and composition using amplification sequencing technology targeting ITS1 region in 52 patients with IBD (34 with Crohn’s disease and 18 with ulcerative colitis), along with 37 healthy controls. Distinctions were observed between healthy controls and individuals with IBD regarding both α diversity and β diversity of microbial communities. Notably, following a 38-week treatment regimen involving anti-TNF-α biologics administration, bacterial communities within patients suffering from IBD exhibited similarities to those found in healthy controls; thus suggesting that anti-TNF therapy may have induced favorable alterations within their microbiome (85). Clinical remission in patients with inflammatory bowel disease (IBD) treated with anti-TNF-α drugs is associated with reduced activity of pro-inflammatory macrophages. Anti-TNF antibodies induce the formation of FC-dependent CD206^+^ regulatory macrophages, which can inhibit activated T cell proliferation and produce anti-inflammatory cytokines to alleviate intestinal inflammation (86). A study involving 11 CD patients undergoing scRNA-seq revealed that gene expression of site-specific CD14^+^CD64^+^CD163^+^ macrophages was specifically linked to treatment failure with anti-TNF-α therapy (87). With advancements in technology, biologics and small molecule drugs developed for IBD are progressing towards fewer side effects and more precise targeting. Various types of biologics have been introduced into clinical practice, including novel inhibitors targeting cytokines (e.g., IL-12/23 inhibitors, PDE4 inhibitors), integrins (e.g., integrin inhibitors), cytokine signaling pathways (e.g., JAK inhibitors, SMAD7 blockers), and cell signaling receptors (e.g., S1P receptor modulators), which have become the preferred treatment options for many IBD patients.

**(2) Fecal microbiota transplantation (FMT)**

FMT is an emerging therapeutic approach that has been developed to effectively manage recurrent Clostridium difficile infections, achieving cure rates of 80–90%. In recent years, it has also demonstrated its potential in the treatment of inflammatory bowel disease (IBD). The primary focus of FMT treatment for IBD lies in targeting patients with mild to moderate ulcerative colitis, aiming to attain clinical remission. However, there is limited evidence supporting the efficacy of FMT in treating Crohn’s disease (CD), although two randomized controlled trials have shown promising results in inducing and maintaining remission (88). Notably, FMT has been found to modulate the composition and diversity of microbiota in colon and fecal samples from UC patients. Specifically, *Fusobacterium gonidiaformans*, *Sutterella wadsworthensis*, and *Escherichia* were enriched while lipopolysaccharide and heme biosynthesis increased among non-remitters. Conversely, *Eubacterium hallii* and *Roseburia inulivorans* were enriched among responders after FMT treatment along with an increase in short-chain fatty acid (SCFA) production and secondary bile acid biosynthesis (89). This may be attributed to the ability of FMT therapy to enhance the abundance of SCFA-producing bacteria (90), which can subsequently exert anti-inflammatory effects on macrophages.

**(3) Probiotics**

Probiotics are live microorganisms that confer health benefits to the host when administered in sufficient quantities. The effects of probiotics on the human body vary depending on the strain, encompassing the production of antibacterial components such as lactic acid, hydroperoxides, and bacteriocins; inhibition of binding sites located in epithelial cells; up-regulation of tight junction molecules in the mucosal barrier; degradation of toxin receptors; alteration of pH levels; and competition for essential nutrients. In inflammatory bowel disease (IBD), probiotics can stimulate antibody production by activating toll-like receptors and promoting differentiation into helper T cell 1, enhance phagocytosis and natural killer cell activity, induce apoptosis in T cells, and stimulate anti-inflammatory cytokine production while reducing pro-inflammatory cytokines (91). Xiao et al. (92), utilizing Bifidobacterium longum strain CECT 7894 to assist in treating IFX-treated dextran sulfate sodium-induced enteritis model in mice discovered that Bifidobacterium longum CECT 7894 may modify α diversity of intestinal flora and increase the relative abundance of *Firmicutes*, *Bacteroidetes*, *Bifidobacteriaceae Ruminaceae*, and *Lachnospiraceas*), the relative abundance of *Lactobacillaceae* and *Bacteroidaceae* while decreasing *Proteobacteria*’s relative abundance *Moraxella erysipelothriaceae Streptococcaceae* to improve efficacy of IFX in DSS-induced mouse enteritis model (92). Moreover, a recent study has developed a novel intracellular hydrogelation technology for the construction of gelated peritoneal macrophages (GPM) and their combination with probiotics to form a novel GPM-ECN complex, which can enhance the intestinal retention time of probiotics through hitchhiking and effectively neutralize inflammatory factors, thereby providing a new therapeutic strategy for inflammatory bowel disease (93).

## 6 Conclusion

The gut microbiota and macrophages play crucial roles in the pathogenesis of IBD, with their reciprocal interactions significantly impacting disease progression. Dysregulation of intestinal macrophages and alterations in gut microbiota composition contribute to the chronic inflammation observed in IBD. Although current treatments effectively manage symptoms, they fail to address the underlying microbial imbalance. Emerging therapies, such as FMT and probiotics, hold promising potential for restoring gut microbiota equilibrium and modulating macrophage responses. However, challenges persist in optimizing these treatments for widespread clinical application. Currently, we are witnessing the era of biologics, with a wide range of biologics and small molecule inhibitors being extensively employed. However, as time progresses, an increasing number of patients with IBD exhibit unresponsiveness to these treatments. The interplay between intestinal flora and macrophages significantly influences the efficacy of biologics, and further investigation is required to determine whether modulation of this interaction can reduce treatment failure rates. Future research should focus on elucidating the precise mechanisms underlying the interaction between gut microbiota and macrophages in IBD while developing personalized microbiome-based therapies. Such an approach has the potential to revolutionize IBD treatment by offering targeted interventions that not only alleviate symptoms but also rectify immunological and microbial dysbiosis at its core. Therefore, we posit that the reprogramming of macrophage metabolism and the identification of specific pathogenic bacteria represent two promising avenues for future research.
